# Bladder paraganglioma: A case of acute respiratory distress syndrome triggered by large bladder tumor transurethral resection with mild symptoms and atypical imaging

**DOI:** 10.1002/iju5.12580

**Published:** 2023-03-03

**Authors:** Toshifumi Takahashi, Tatsuya Hazama, Hideto Ota, Yuya Yamada, Masakazu Nakashima, Masahiro Tamaki, Noriyuki Ito

**Affiliations:** ^1^ Department of Urology Japanese Red Cross Wakayama Medical Center Wakayama Japan

**Keywords:** ARDS, bladder paraganglioma, TURBT

## Abstract

**Introduction:**

Bladder paraganglioma is exceedingly rare, accounting for <0.05% of bladder tumors. This is a case of paraganglioma with no symptom other than palpitations during urination, with atypical imaging, resulting in acute respiratory distress syndrome after transurethral resection of the bladder tumor.

**Case presentation:**

A 46‐year‐old man underwent transurethral resection of the bladder tumor for a bladder tumor 61 × 52 mm in size on contrast‐enhanced computed tomography. The patient only had micturition attacks and was suspected to have urothelial carcinoma on magnetic resonance imaging. The patient had acute respiratory distress syndrome after the operation which improved conservatively. The ^123^Iodine metaiodobenzylguanidine scintigraphy, urinalysis, and pathological examination revealed bladder paraganglioma. Robot‐assisted radical cystectomy and ileal neobladder reconstruction were performed.

**Conclusion:**

This study reported bladder paraganglioma with no symptoms other than micturition attacks in which acute respiratory distress syndrome occurred after transurethral resection of the bladder tumor.

**Abbreviations & Acronyms**
ARDS: acute respiratory distress syndrome
CECT: contrast‐enhanced computed tomography
GAPP: grading of adrenal pheochromocytoma and paraganglioma
MRI: magnetic resonance imaging
TURBT: transurethral resection of the bladder tumor
WI: weighted imaging

**Keynote message**

It is important to preoperatively diagnose bladder paraganglioma even if the symptoms are mild and imaging atypical as bladder tumor transurethral resection may result in acute respiratory distress syndrome.

## Introduction

Paragangliomas are neural crest‐derived extra‐adrenal neoplasms. Bladder paragangliomas are exceedingly rare, accounting for 10% of paragangliomas and <0.05% of bladder tumors.^1^ A few cases of functional yet asymptomatic bladder paraganglioma and those with atypical images have also been reported.^2^, ^3^

Here, a paraganglioma case with no symptoms other than micturition attacks and atypical imaging was reported, resulting in ARDS after TURBT.

## Case presentation

**Fig. 1:**
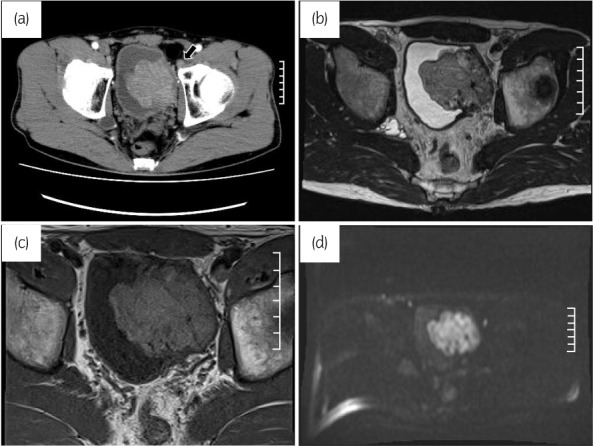
(a) CT revealed a 61 × 52 mm bladder tumor at the left side wall of the bladder and mild enlargement of the left external iliac lymph node (arrow). (b–d) MRI revealed a faint high signal on T2‐WI (b) and a high signal on T1‐WI (c) and DWI (d), which suggested extramural invasion.

**Fig. 2:**
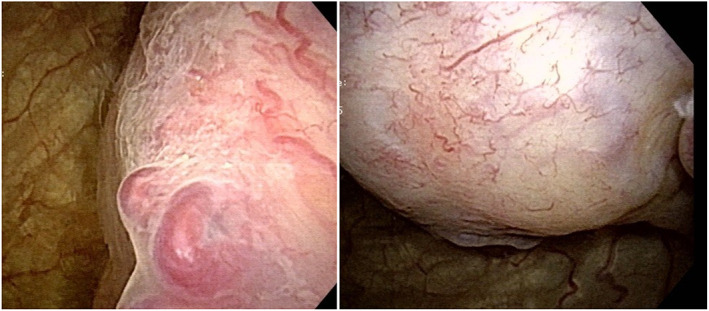
Cystoscopy revealed a non‐papillary smooth surface tumor protruding from the left side wall.

A 46‐year‐old man presented to his physician with anal pain and was found to have a perianal abscess and bladder mass by CECT. After perianal abscess incision and drainage, the patient was referred to our hospital for further bladder mass examination and treatment. Palpitations during urination were observed a few months ago but had no other medical history. Complete blood count and biochemistry tests were within normal ranges; urinalysis showed no hematuria, and urinary cytology showed a class III lesion. CECT showed a 61 × 52 mm bladder tumor at the left bladder sidewall and mild left external iliac lymph node enlargement. MRI showed a faint high signal intensity on T2‐WI and a high signal intensity on T1‐WI and DWI, which suggested urothelial carcinoma with extramural invasion (Fig. 1). Cystoscopy showed a non‐papillary submucosal tumor protruding from the left sidewall (Fig. 2). Because it was atypical as bladder paraganglioma from the MRI image and there was no symptom other than recent micturition attacks, muscle‐invasive bladder cancer was diagnosed, and then TURBT was performed. Systolic blood pressure increased to 240 mmHg when the bladder tumor was resected. The tumor was resected in small volumes to confirm the histological types, reducing the operation time. However, the patient complained of dyspnea after leaving the operating room. Percutaneous oxygen saturation fell to 86% with 3 L of oxygen administration and chest CT showed ground‐glass opacities on the dorsal side of the bilateral lung fields, which led to the ARDS diagnosis. The symptom improved after oxygen administration, and oxygen administration could be discontinued 1 day postoperatively.

**Fig. 3:**
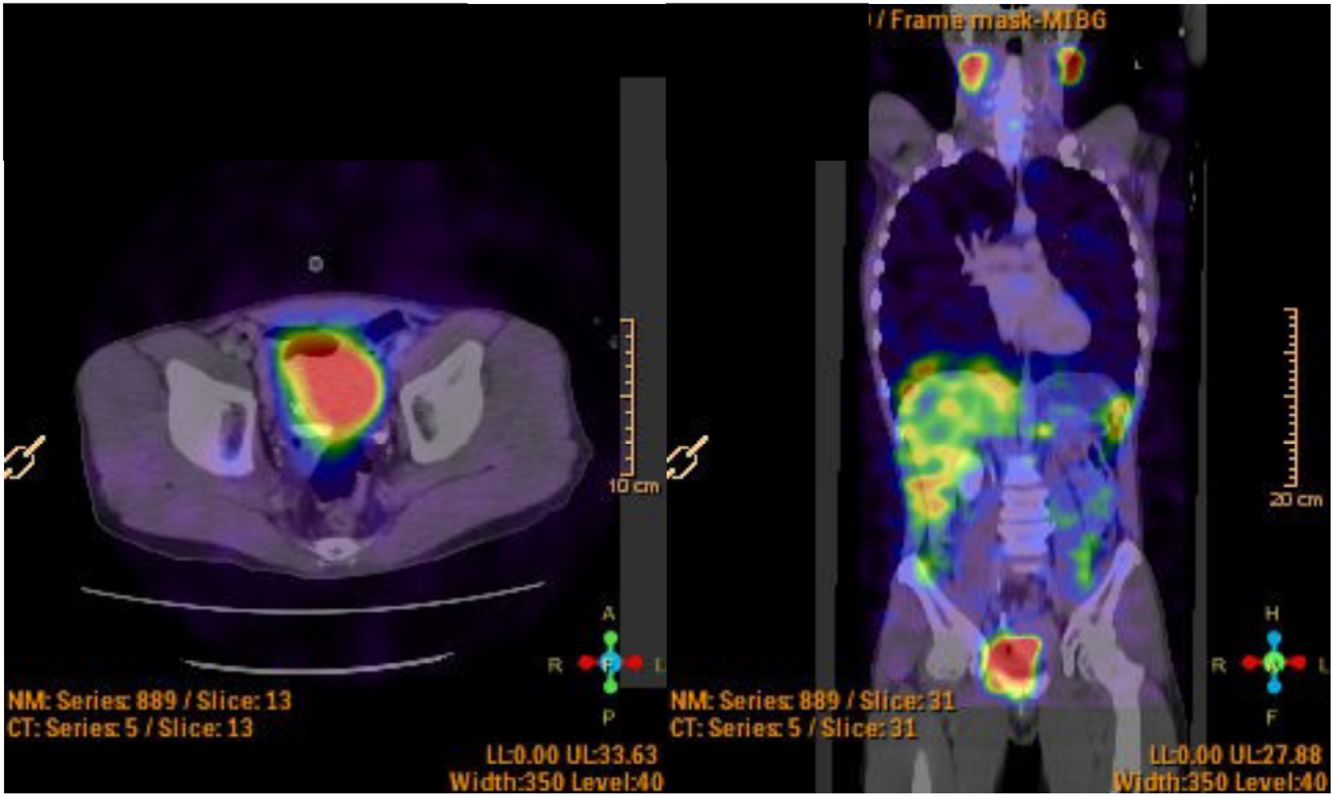
The ^123^I‐MIBG scintigraphy revealed accumulation consistent with a bladder mass.

The ^123^I‐MIBG scintigraphy showed accumulation consistent with a bladder mass (Fig. 3). Serum catecholamines and 24‐h urine catecholamines, metanephrine, and vanillylmandelic acid levels were elevated above the normal range.

**Fig. 4:**
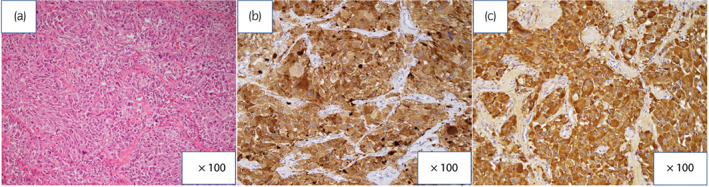
(a) Hematoxylin–eosin staining showed that polygonal and amphophilic cells proliferated in alveolar or trabecular patterns and were arranged in large and irregular cell nests. An immunohistochemical study showed positive findings for (b) synaptophysin and (c) chromogranin A.

Pathological analysis revealed that polygonal and amphophilic cells proliferated in alveolar or trabecular patterns, arranged in large and irregular cell nests on hematoxylin–eosin staining. Cellularity was moderate. An immunohistochemical study revealed positive findings for chromogranin A, synaptophysin, and Ki‐67 (<1%) (Fig. 4). Based on the above findings, the pathological diagnosis was urinary bladder paraganglioma. The GAPP score was 2.

After oral doxazosin mesylate administration, the dose gradually increased to 16 mg/day for preoperative preparation. Robot‐assisted radical cystectomy and ileal neobladder reconstruction were performed. The left external iliac lymph node was smaller than before on the CT scan just before surgery. Intraoperative rapid pathological diagnosis at the equivalent site showed no evidence of malignancy. Thus, pelvic lymphadenectomy was omitted. Systolic blood pressure sometimes rose to 180 mmHg during surgery and phentolamine was administered at 2–10 mg/hour as a continuous intravenous and 1–2 mg as a bolus injection when appropriate, but after tumor removal, it remained in the normal range and no special vasopressors or antihypertensives were used. On histopathologic analysis, the bladder paraganglioma showed invasion into the perivesical fat, but the resection margins were negative. The patient had an uneventful postoperative clinical course and was discharged on postoperative day 22. Postoperatively, serum and urinary catecholamine levels normalized. Genetic testing was not performed in this case without the patient's request.

## Discussion

Urinary bladder paraganglioma are relatively rare tumors and have been reported to account for <0.05% of bladder tumors.^1^ Symptoms do not appear in all cases, and it has been reported that symptoms, such as hypertension, headache, and micturition attacks associated with increased catecholamine production occur in 50% of cases.^4^ Here, the lack of typical symptoms and atypical MRI findings led to the belief that bladder paraganglioma was unlikely. Therefore, TUR was performed, which resulted in postoperative ARDS development.

The most common signs and symptoms of bladder paraganglioma are micturition attacks, hypertension, headache, and hematuria.^4^ Yuan et al. reported that functional bladder paraganglioma with endocrine activity were 28.6% in tumors <3 cm in diameter and 78% in tumors >3 cm, suggesting that larger tumors may be functional bladder paragangliomas.^5^ However, about 10% of functional bladder paraganglioma with endocrine activity do not show symptoms.^2^ It has been reported that 38.9% of patients with bladder paraganglioma were diagnosed preoperatively in Japan.^6^ Kurose et al. noted the importance of young age, hypertension, micturition attacks, the number of symptoms, and large tumor size in diagnosing bladder paraganglioma, with micturition attacks being particularly useful. One report found 69% postoperative complications in patients without preparation for pheochromocytoma,^7^ while the other reported 8.9% postoperative pulmonary complications in patients with preparation for pheochromocytoma or paraganglioma.^8^ TUR for bladder paraganglioma is contraindicated in principle because it may induce hypertension and tachycardia because of intraoperative manipulation and may cause heart failure.^1^ Although there have been no reports of ARDS caused by TUR for bladder paraganglioma, there have been some reports of severe hypertension, so the possibility of hypertensive acute heart failure and ARDS may not be low indeed.^3^, ^9^ Therefore, it is crucial to suspect and diagnose bladder paraganglioma preoperatively to avoid TUR, which may cause heart failure and ARDS as shown in this case.

In addition to cystoscopy and echography, diagnostic imaging for bladder paraganglioma includes CT, MRI imaging, and nuclear medicine scans, such as ^123^I‐MIBG scintigraphy and ^18^F‐fluorodeoxyglucose‐position emission tomography. MRI shows low‐signal intensity on T1‐WI and moderately high‐signal intensity on T2‐WI typically; sensitivity can be as high as 88%.^10^ The ^123^I‐MIBG scintigraphy also has a high specificity of 96%.^7^ However, in cases such as this one, MRI sometimes does not show typical images and a case of bladder paraganglioma with negative MIBG scintigraphy has also been reported.^3^ Therefore, it is assumed that it was challenging to rule out bladder paraganglioma beforehand based on atypical image findings.

A systematic review by Beilan et al. reported that 68.9% of patients who underwent surgery for bladder paraganglioma underwent partial cystectomy, 11.3% underwent total cystectomy, and 19.8% underwent TURBT as surgical procedures for bladder paraganglioma.^4^ Overall, postoperative recurrence was observed in 14.2% and metastasis in 9.4%, according to the report.^4^ Complete tumor resection is desirable to prevent a postoperative recurrence. In this case, total cystectomy rather than partial cystectomy was selected because MRI findings showed a large tumor, 61 × 52 mm in size, and extramural invasion which showed invasion into the perivesical fat on histopathologic analysis as a result, and partial cystectomy might not have achieved complete resection.

In conclusion, this study reported a bladder paraganglioma with no symptoms other than micturition attacks, in which ARDS occurred after TURBT.

## Author contributions

Toshifumi Takahashi: Data curation; formal analysis; supervision; validation; visualization; writing – original draft. Tatsuya Hazama: Data curation. Hideto Ota: Data curation. Yuya Yamada: Data curation. Masakazu Nakashima: Data curation; supervision; validation. Masahiro Tamaki: Data curation; supervision; validation. Noriyuki Ito: Data curation; supervision.

## Approval of the research protocol by an institutional reviewer board

Not applicable.

## Informed consent

Written informed consent to participate in this study and for the publication of this report was obtained from the patient for ethics approval.

## Registry and the registration No. of the study/trial

Not applicable.

## References

[1] Dahm P , Gschwend JE . Malignant non‐urothelial neoplasms of the urinary bladder: a review. Eur. Urol. 2003; 44: 672–81. 14644119 10.1016/s0302-2838(03)00416-0

[2] Messerli F , Finn M , Macphee A . Pheochromocytoma of the urinary bladder. Systemic hemodynamics and circulating catecholamine levels. JAMA 1982; 247: 1863–4. 7062490

[3] Sugimura R , Kawahara T , Noguchi G *et al*. Functional paraganglioma of the bladder: both radiographic‐negative and laboratory‐negative case. IJU Case Rep. 2019; 2: 174–7. 32743403 10.1002/iju5.12071 PMC7292094

[4] Beilan JA , Lawton A , Hajdenberg J , Rosser C . Pheochromocytoma of the urinary bladder: a systematic review of the contemporary literature. BMC Urol. 2013; 13: 22. 23627260 10.1186/1471-2490-13-22 PMC3654956

[5] Yuan Y , Su Z , Zhu R , Li X , Xu G . Bladder paraganglioma: three cases report and literature review. Int. Med. Case Rep. J. 2021; 14: 765–71. 34803407 10.2147/IMCRJ.S336659 PMC8594893

[6] Kurose H , Ueda K , Uegaki M *et al*. Paraganglioma of the urinary bladder: case report and literature reviews. IJU Case Rep. 2020; 3: 192–5. 32914072 10.1002/iju5.12185 PMC7469756

[7] Goldstein RE , O'Neill JA Jr , Holcomb GW III *et al*. Clinical experience over 48 years with pheochromocytoma. Ann. Surg. 1999; 229: 755–64. 10363888 10.1097/00000658-199906000-00001 PMC1420821

[8] Weingarten TN , Welch TL , Moore TL *et al*. Preoperative levels of catecholamines and metanephrines and intraoperative hemodynamics of patients undergoing pheochromocytoma and paraganglioma resection. Urology 2017; 100: 131–8. 27769919 10.1016/j.urology.2016.10.012

[9] Iwamoto G , Kawahara T , Tanabe M *et al*. Paraganglioma in the bladder: a case report. J. Med. Case Rep. 2017; 11: 306. 29084607 10.1186/s13256-017-1473-2 PMC5663097

[10] Wong‐You‐Cheong J , Woodward P , Manning M , Sesterhenn I . From the archives of the AFIP: neoplasms of the urinary bladder: radiologic‐pathologic correlation. Radiographics 2006; 26: 553–80. 16549617 10.1148/rg.262055172

